# Supplementary material for: Structural Basis for the Regiospecificity of a Lipase from Streptomyces sp. W007
Source: Int J Mol Sci. 2022 May 22;23(10):5822. doi: 10.3390/ijms23105822 (PMC9146090; doi:10.3390/ijms23105822)
Supplement: Supplementary file 1 [file ijms-23-05822-s001.zip › ijms-1738550-supplementary.pdf]

Supporting Information

Structural basis for the regiospecificity of a lipase from *Streptomyces* sp. W007

Zexin Zhao[1], Siyue Chen[2], Long Xu[3], Jun Cai[1], Jia Wang[4, *] & Yonghua Wang[2, 5, *]

[1] Key Laboratory of Fermentation Engineering (Ministry of Education), Hubei Key Laboratory of Industrial Microbiology, Hubei University of Technology, Wuhan 430068, PR China

[2] School of Food Science and Engineering, South China University of Technology, Guangzhou 510640, PR China

[3] College of Food Science and Technology, Henan Agricultural University, Zhengzhou 450002, PR China

[4] College of Life Science, Guangzhou University, Guangzhou 510006, PR China

[5] Guangdong Youmei Institute of Intelligent Bio-manufacturing, Foshan 528225, PR China

* To whom correspondence should be addressed:

Prof. Yonghua Wang E-Mail: yonghw@scut.edu.cn

Dr. Jia Wang E-mail: jiawang@gzhu.edu.cn

Supplementary Figures & Tables

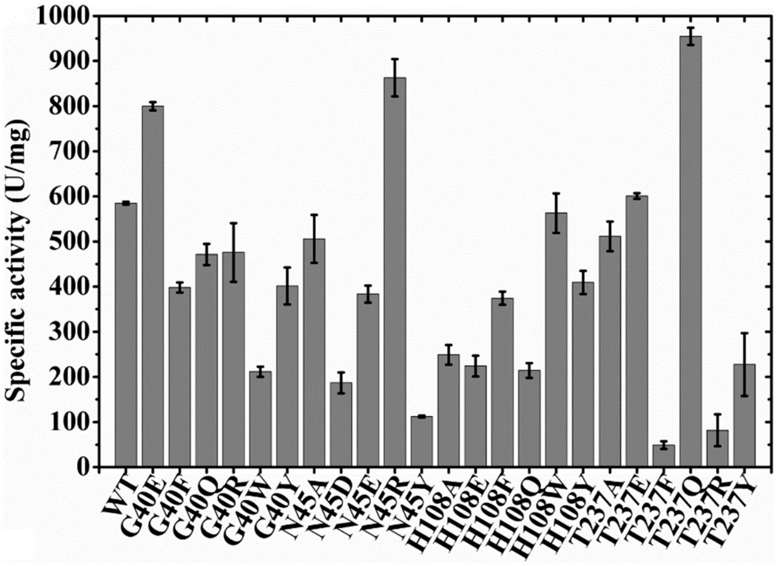

Figure S1. Specific activity of MAS1 and its mutants.

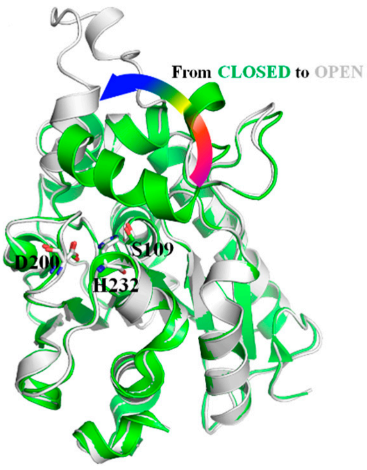

Figure S2. The superimposition of MAS1 in closed and open conformation.

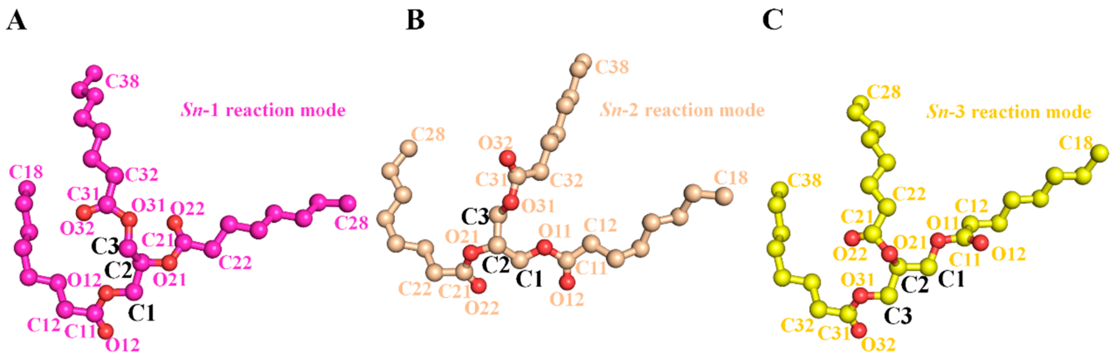

Figure S3. Atom numbering of TC in the **(A)** *sn*-1, **(B)** *sn*-2 and **(C)** *sn*-3 reaction modes, respectively.

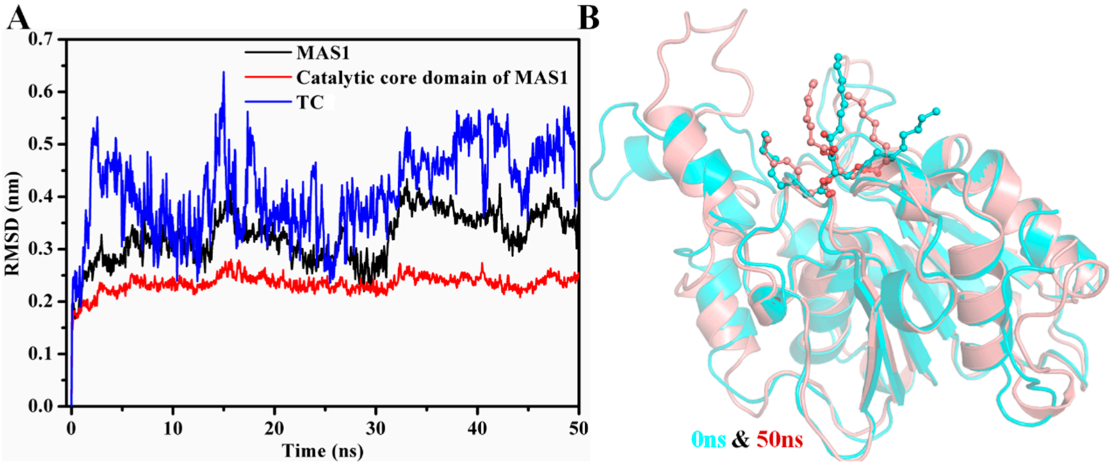

Figure S4. The analysis of MD simulation of MAS1. **(A)** The RMSD variation analysis. **(B)** Overall structural comparison between the initial and final models.

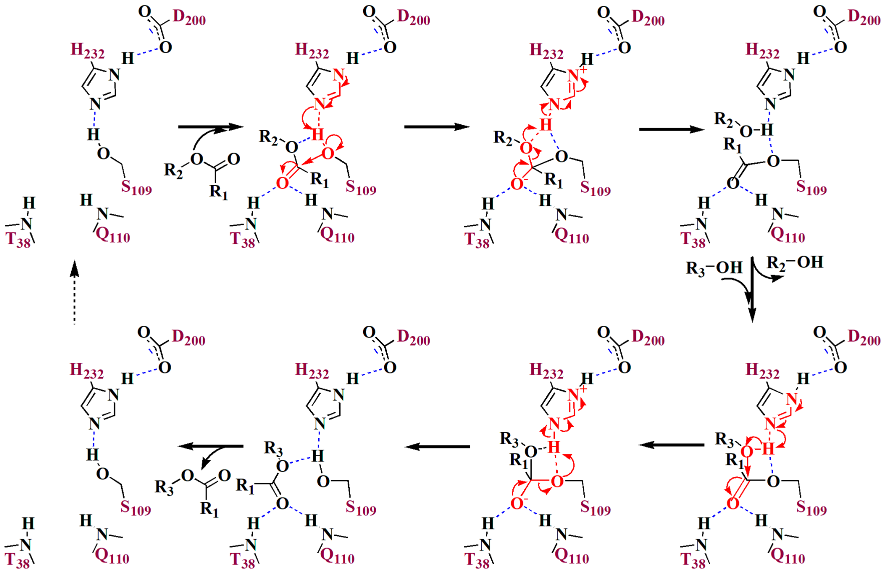

Figure S5. The reaction mechanism of acyl transformation catalyzed by MAS1 (It was hydrolysis process, when R_3 was set as H).

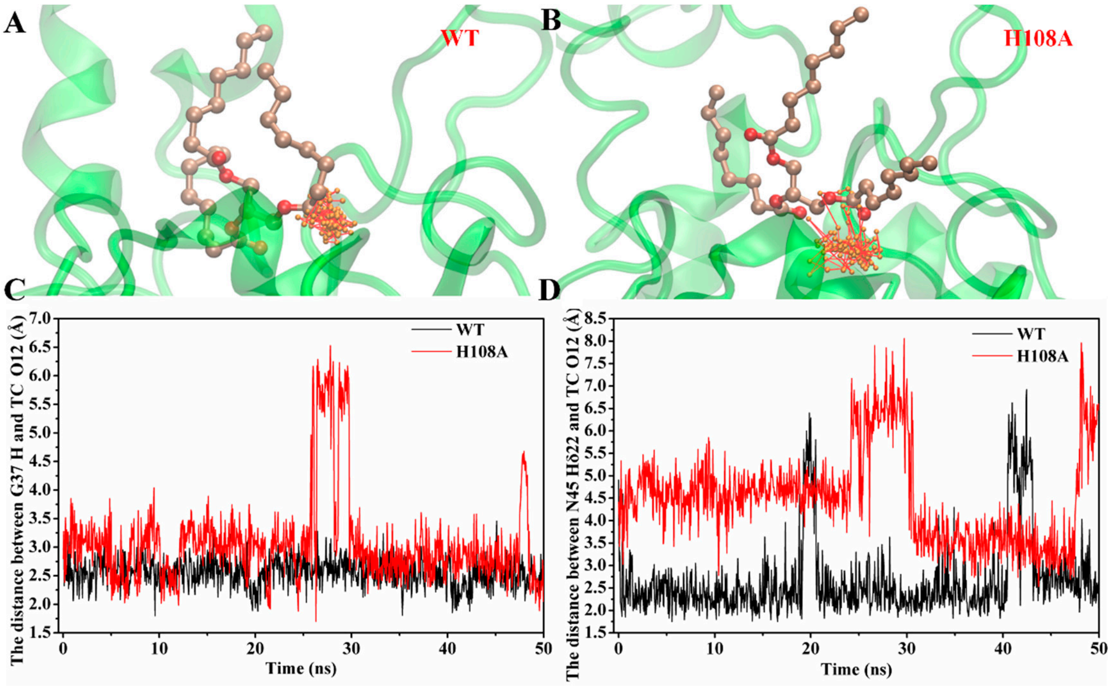

Figure S6. The comparison of binding stability of MAS1 and H108A with *sn*-1 moiety of TC. The track of TC O12 in **(A)** wild-type and **(B)** H108A models. The track was presented as gold balls connected by red line. **(C-D)** The distance fluctuation analysis.

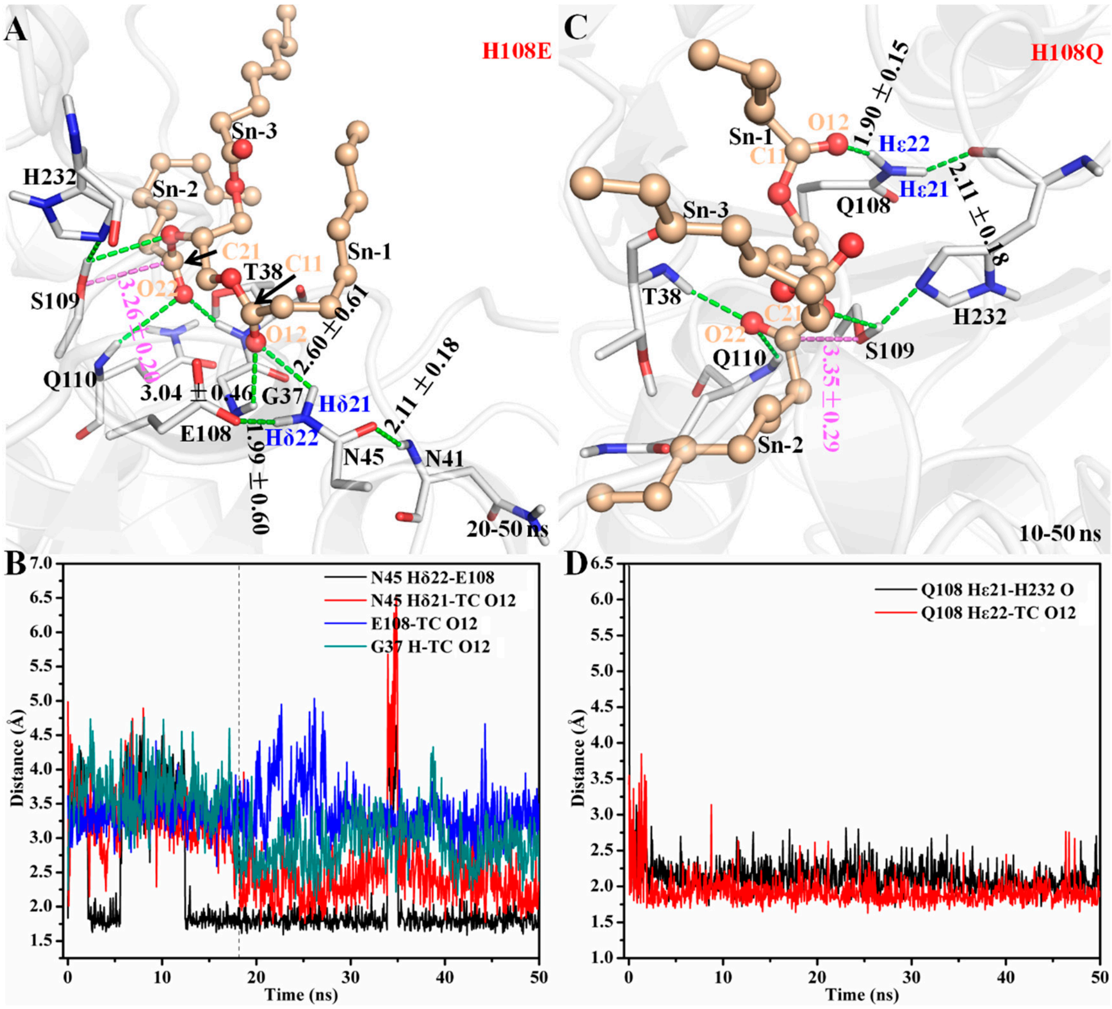

Figure S7. The comparison of computational results of mutants H108E and H108Q. The substrate binding conformation of mutants **(A)** H108E and **(C)** H108Q model in balanced phase of MD simulation. The distance fluctuation analysis of mutants **(B)** H108E and **(D)** H108Q.

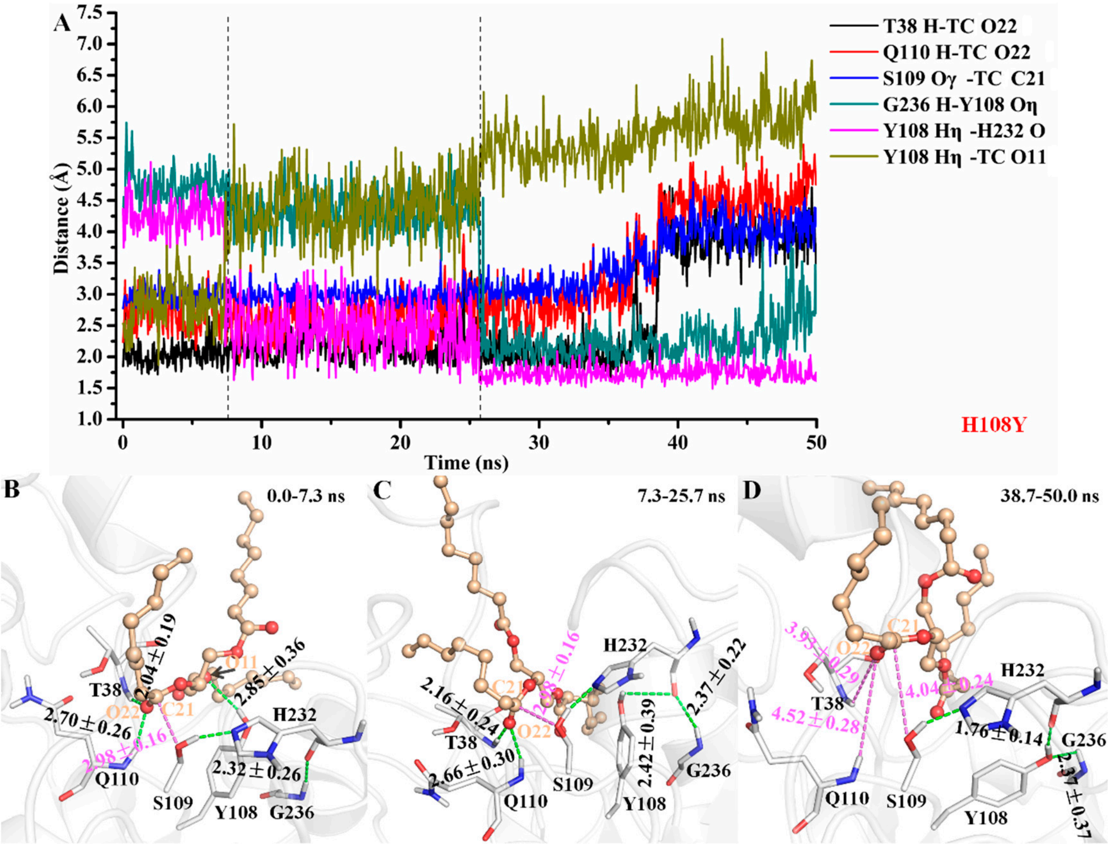

Figure S8. The computational results of mutant H108Y. **(A)** The distance fluctuation analysis. **(B-D)** Substrate binding conformation in different phases of MD simulation.

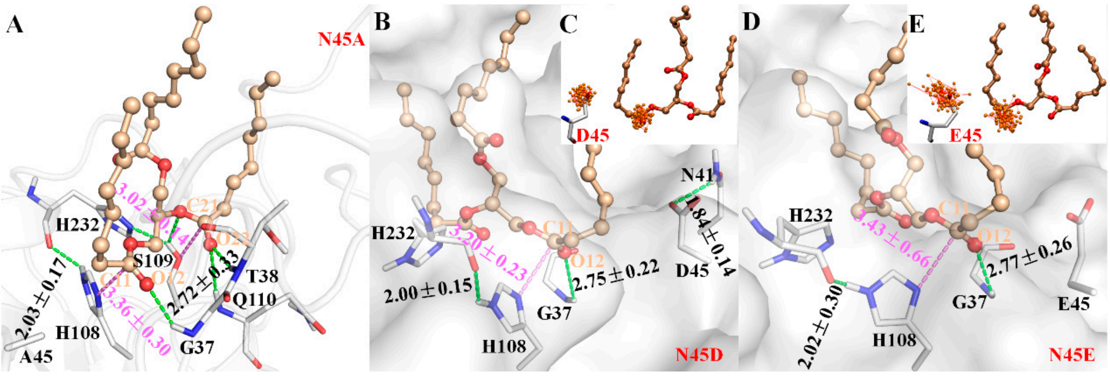

Figure S9. The comparison of computational results of mutants N45A, N45D and N45E. The substrate binding conformation of mutants **(A)** N45A, **(B)** N45D and **(D)** N45E. The trajectory of TC O12 and carboxyl carbon of introduced residue in mutants **(C)** N45D and **(E)** N45E. The trajectory was presented as gold balls connected by red line.

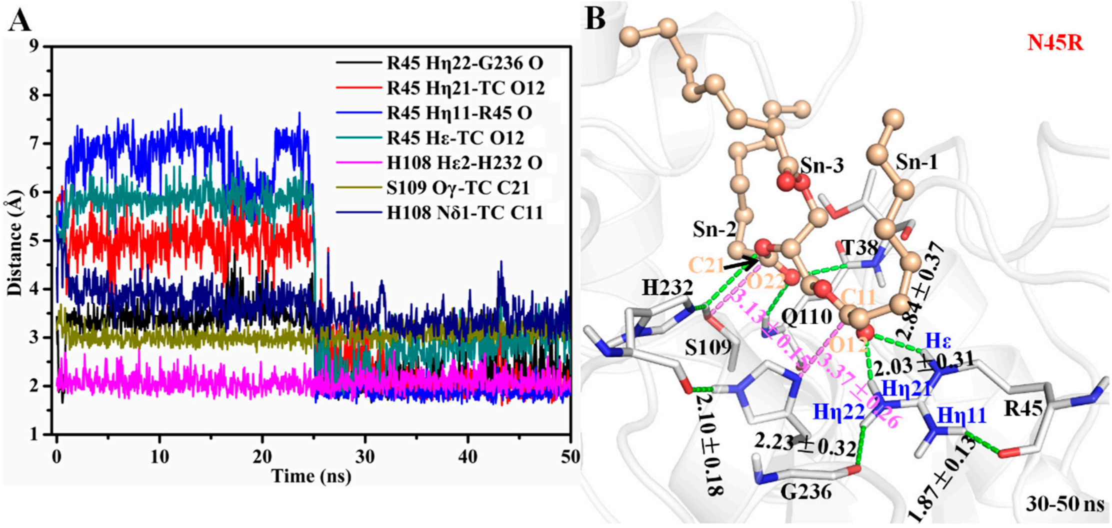

Figure S10. The computational results of mutant N45R. **(A)** The distance fluctuation analysis. **(B)** Substrate binding conformation in balanced phases of MD simulation.

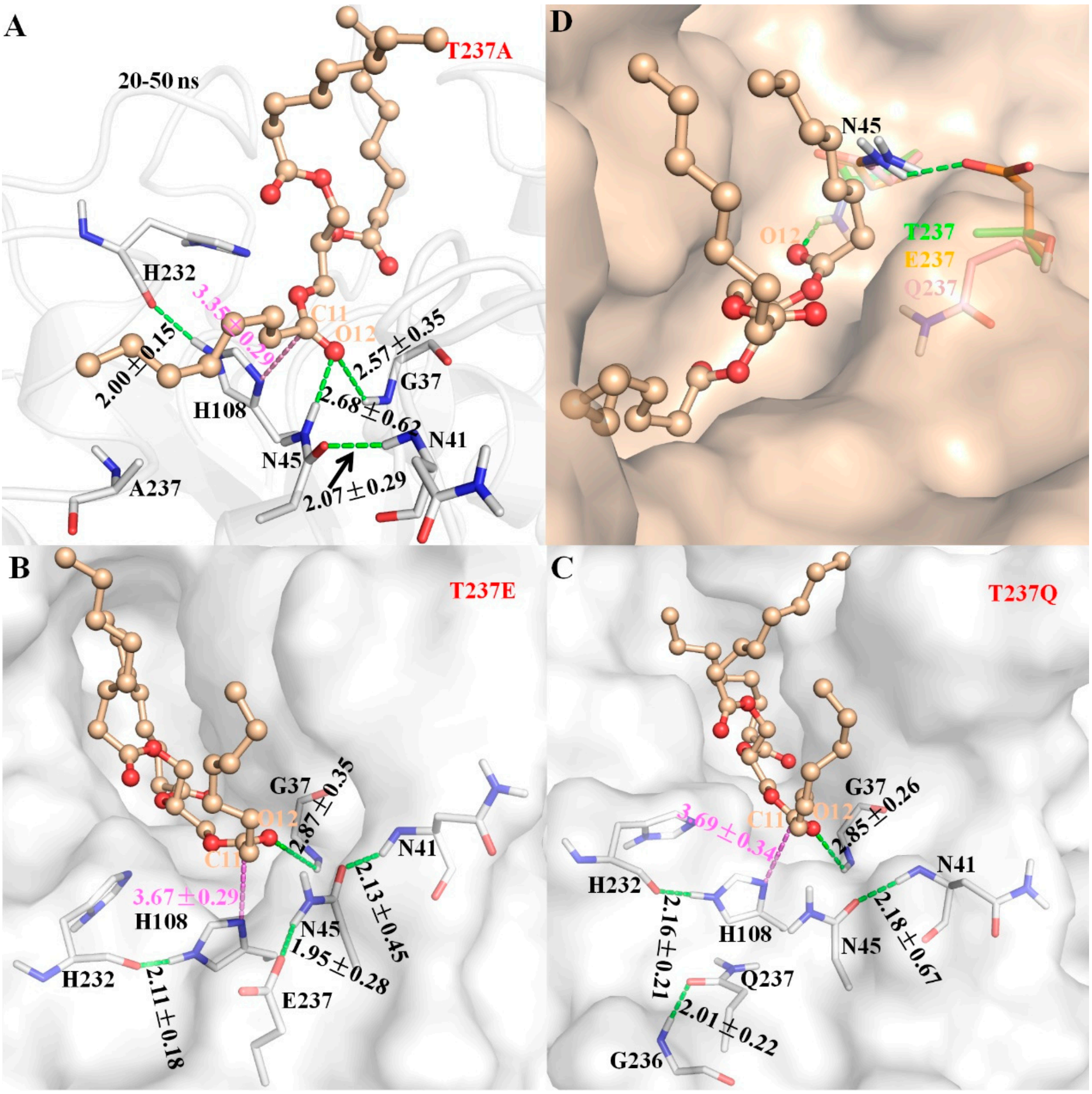

Figure S11. The comparison of computational results of mutants T237A, T237E and T237Q. The substrate binding conformation of mutants **(A)** T237A, **(B)** T237E and **(C)** T237Q. **(D)** The structural alignment of wild-type MAS1 and its mutants T237E and T237Q. The wild type was showed as wheat surface representation.

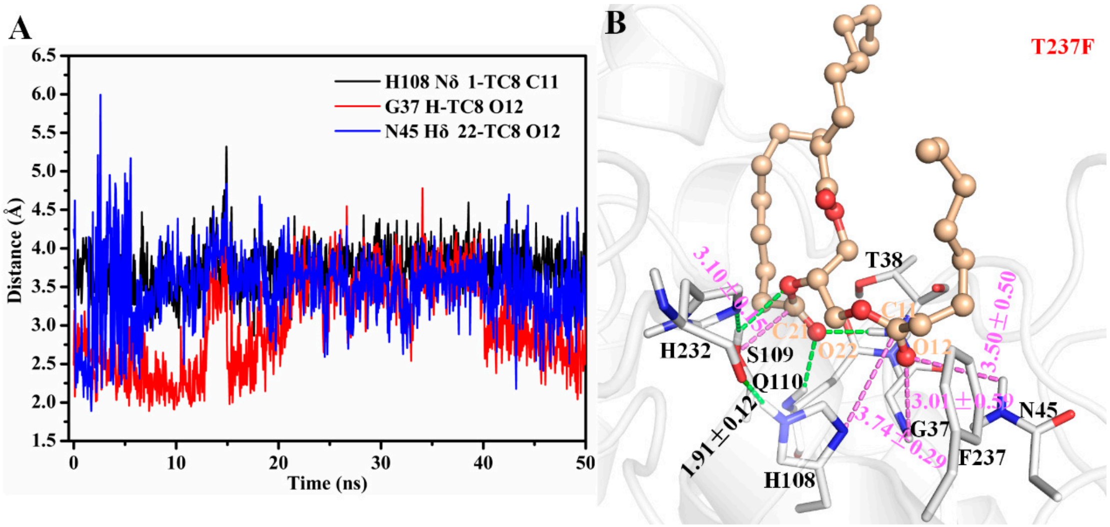

Figure S12. The computational results of mutant T237F. **(A)** The distance fluctuation analysis. **(B)** Substrate binding conformation during MD simulation.

Table S1. Primers list of vector exchange and mutants construction.

Primer name	Sequence(5'→3')
Pet22b-mas1_F[a]	TTAATTCGGATCCGAATTCGGCCACGCCAGCTGCTGAG
Pet22b-mas1_R[a]	TGGTGGTGGTGGTGCTCGAGGCCAATCACAGAAGCACAGGTT
G40E_F	GTACATGGCACCTTCGAGAACTCAATTGACAACTG
G40E_R	CAGTTGTCAATTGAGTTCTCGAAGGTGCCATGTAC
G40F_F	CTTGTACATGGCACCTTCTTTAACTCAATTGACAACTGG
G40F_R	CCAGTTGTCAATTGAGTTAAAGAAGGTGCCATGTACAAG
G40Q_F	CTTGTACATGGCACCTTCCAAAACTCAATTGACAACTGGC
G40Q_R	GCCAGTTGTCAATTGAGTTTTGGAAGGTGCCATGTACAAG
G40R_F	AGTTGTCAATTGAGTTACGGAAGGTGCCATGTACAAG
G40R_R	CTTGTACATGGCACCTTCCGTAACTCAATTGACAACT
G40W_F	TCTTGTACATGGCACCTTCTGGAACTCAATTGACAACTGGC
G40W_R	GCCAGTTGTCAATTGAGTTCCAGAAGGTGCCATGTACAAGA
G40Y_F	CTTGTACATGGCACCTTCTATAACTCAATTGACAACTGG
G40Y_R	CCAGTTGTCAATTGAGTTATAGAAGGTGCCATGTACAAG
N45A_F	CTTCGGTAACTCAATTGACGCCTGGCTTGTTTTGGCTCCA
N45A_R	TGGAGCCAAAACAAGCCAGGCGTCAATTGAGTTACCGAAG
N45D_F	GAGCCAAAACAAGCCAGTCGTCAATTGAGTTACCGAA
N45D_R	TTCGGTAACTCAATTGACGACTGGCTTGTTTTGGCTC
N45E_F	ATGGAGCCAAAACAAGCCACTCGTCAATTGAGTTACCGAAG
N45E_R	CTTCGGTAACTCAATTGACGAGTGGCTTGTTTTGGCTCCAT
N45F_F	TGGAGCCAAAACAAGCCAGAAGTCAATTGAGTTACCGAAG
N45F_R	CTTCGGTAACTCAATTGACTTCTGGCTTGTTTTGGCTCCA
N45R_F	GCCAAAACAAGCCACCTGTCAATTGAGTTACCGAAGGTGCCA
N45R_R	TGGCACCTTCGGTAACTCAATTGACAGGTGGCTTGTTTTGGC
N45Y_F	CCTTCGGTAACTCAATTGACTATTGGCTTGTTTTGGCTCCATA
N45Y_R	TATGGAGCCAAAACAAGCCAATAGTCAATTGAGTTACCGAAGG
S109A_F	GATCTGGTCGGTCACGCCCAAGGTGGTATGATG
S109A_R	CATCATACCACCTTGGGCGTGACCGACCAGATC
H108A_F	CAAGGCTGATCTGGTCGGTGCCAGTCAAGGTGGTATGATG
H108A_R	CATCATACCACCTTGACTGGCACCGACCAGATCAGCCTTG
H108E_F	CATACCACCTTGACTCTCACCGACCAGATCAGCCT
H108E_R	AGGCTGATCTGGTCGGTGAGAGTCAAGGTGGTATG
H108F_F	CATCATACCACCTTGACTGAAACCGACCAGATCAGCCTTG
H108F_R	CAAGGCTGATCTGGTCGGTTTCAGTCAAGGTGGTATGATG
H108Q_F	CCACCTTGACTCTGACCGACCAGATCAGCC
H108Q_R	GGCTGATCTGGTCGGTCAGAGTCAAGGTGG
H108R_F	CATACCACCTTGACTGCGACCGACCAGATCAGC
H108R_R	GCTGATCTGGTCGGTCGCAGTCAAGGTGGTATG
H108W_F	CAAGGCTGATCTGGTCGGTTGGAGTCAAGGTGGTATGATG
H108W_R	CATCATACCACCTTGACTCCAACCGACCAGATCAGCCTTG
H108Y_F	CATCATACCACCTTGACTATAACCGACCAGATCAGCCTTGG
H108Y_R	CCAAGGCTGATCTGGTCGGTTATAGTCAAGGTGGTATGATG
H232A_F	GTGGATTTGTCAGAAGCCGTAGCCATCGGAAC
H232A_R	GTTCCGATGGCTACGGCTTCTGACAAATCCAC
T237A_F	AGCTATCCTATCGATAGCTCCGATGGCTACGTGTT
T237A_R	AACACGTAGCCATCGGAGCTATCGATAGGATAGCT
T237E_F	GAACACGTAGCCATCGGAGAAATCGATAGGATAGCTTTTC
T237E_R	GAAAAGCTATCCTATCGATTTCTCCGATGGCTACGTGTTC
T237F_F	GAAAAGCTATCCTATCGATAAATCCGATGGCTACGTGTTCTG
T237F_R	CAGAACACGTAGCCATCGGATTTATCGATAGGATAGCTTTTC
T237Q_F	GAACACGTAGCCATCGGACAAATCGATAGGATAGCTTTTC
T237Q_R	GAAAAGCTATCCTATCGATTTGTCCGATGGCTACGTGTTC
T237R_F	AAAGCTATCCTATCGATCCTTCCGATGGCTACGTGTTCTGA
T237R_R	TCAGAACACGTAGCCATCGGAAGGATCGATAGGATAGCTTT
T237Y_F	GAACACGTAGCCATCGGATACATCGATAGGATAGCTTTTC
T237Y_R	GAAAAGCTATCCTATCGATGTATCCGATGGCTACGTGTTC

[a] The homologous region of vector exchange primers were marked in red and the

restriction sites were underlined.
